# Metalloprotease Adam10 suppresses epilepsy through repression of hippocampal neuroinflammation

**DOI:** 10.1186/s12974-018-1260-z

**Published:** 2018-08-04

**Authors:** Xinjian Zhu, Xiaolin Li, Mengyi Zhu, Kangni Xu, Li Yang, Bing Han, Rongrong Huang, Aifeng Zhang, Honghong Yao

**Affiliations:** 1 Department of Pharmacology, Medical School of Southeast University, Dingjiaqiao 87th, Nanjing, 210009 China; 2 Department of Geriatrics, The First Affiliated Hospital of Nanjing Medical University, Nanjing, China; 3 Department of Pathology, Medical School of Southeast University, Nanjing, China

**Keywords:** Metalloprotease, Adam10, Hippocampus, Neuroinflammation, Temporal lobe epilepsy

## Abstract

**Background:**

Mice with pilocarpine-induced temporal lobe epilepsy (TLE) are characterized by intense hippocampal neuroinflammation, a prominent pathological hallmark of TLE that is known to contribute to neuronal hyperexcitability. Recent studies indicate that Adam10, a member of a disintegrin and metalloproteinase domain-containing protein (Adam) family, has been involved in the neuroinflammation response. However, it remains unclear whether and how Adam10 modulates neuroinflammation responses in the context of an epileptic brain or whether Adam10 affects epileptogenesis via the neuroinflammation pathway.

**Methods:**

Adult male C57BL/6J mice were subjected to intraperitoneal injection of pilocarpine to induce TLE. Adeno-associated viral (AAV) vectors carrying Adam10 (AAV-Adam10) or lentiviral vectors carrying short hairpin RNA, which is specific to the mouse Adam10 mRNA (shRNA-Adam10), were bilaterally injected into the hippocampus to induce overexpression or knockdown of Adam10, respectively. The specific anti-inflammatory agent minocycline was administered following status epilepticus (SE) to block hippocampal neuroinflammation. Continuous video EEG recording was performed to analyze epileptic behavior. Western blot, immunofluorescence staining, and ELISA were performed to determine Adam10 expression as well as hippocampal neuroinflammation.

**Results:**

In this study, we demonstrate that overexpression of Adam10 in the hippocampus suppresses neuroinflammation and reduces seizure activity in TLE mice, whereas knockdown of Adam10 exacerbates hippocampal neuroinflammation and increases seizure activity. Furthermore, increased seizure activity in Adam10 knockdown TLE mice is dependent on hippocampal neuroinflammation.

**Conclusion:**

These results suggest that Adam10 suppresses epilepsy through repression of hippocampal neuroinflammation. Our findings provide new insights into the Adam10 regulation of development of epilepsy via the neuroinflammation pathway and identify a potential therapeutic target for epilepsy.

## Background

Adam10 is a member of the ADAM metalloprotease family and is able to cleave the extracellular domains of several membrane-bound proteins in a process called ectodomain shedding [1–3]. One of the major substrates of Adam10 is amyloid precursor protein (APP), for which Adam10 acts as an α-secretase to prevent the excessive production of the pathogenic amyloid β (Aβ) peptide [4, 5], a hallmark of Alzheimer’s disease (AD). The processing of APP by Adam10 produces a soluble N-terminal APP fragment (sAPP), which has been shown to exert neurotrophic and neuroprotective effects [6]. Thus, the activation of Adam10 has been suggested as a therapeutic approach for AD patients [4, 7]. Despite the crucial role of Adam10 in AD, recent studies indicate that Adam10 may contribute to other neurological and psychiatric disease. A previous study reported that postnatal disruption of Adam10 in the brain causes epileptic seizures, learning deficits, altered neuronal spine morphology, and defective synaptic functions [8], suggesting that Adam10 plays a pivotal role in the synaptic and neuronal network activity. This finding is supported by evidence that conditional Adam10^−/−^ mice exhibit mistargeted axons and a dysregulated neuronal network [9]. Additionally, Adam10 expression has been found to be altered in the dentate gyrus of kainic acid-induced epileptic rats [10], indicating an association of Adam10 with epilepsy. It is generally accepted that neuroinflammation is a prominent pathological hallmark of TLE, which is known to contribute to neuronal hyperexcitability in both human patients and animal models [11–14]. These studies indicate that seizure-induced proinflammatory signals may play a pivotal role in recurrent epilepsy. Adam10 has been largely distributed in the astrocytes [15, 16], as well as neurons [17], and it has been found to be responsible for proteolytic processing of CX3CL1, a chemokine primarily expressed in the neurons and astrocytes, which is involved in the neuroinflammation response [16]. However, it remains unclear whether and how Adam10 modulates the neuroinflammatory response in the context of an epileptic brain or whether Adam10 affects epileptogenesis via the neuroinflammation pathway. Thus, in the present study, we sought to explore the role of Adam10 in neuroinflammation of the epileptic brain and to further determine whether Adam10 affects epileptogenesis through neuroinflammation pathways.

## Methods

### Animals

Male C57BL/6J mice (4–6 weeks old; weighing 19 ± 2 g at the beginning of the experiments) were obtained from Nanjing Biomedical Research Institute of Nanjing University (NBRI) (Nanjing, China). The animals were housed in plastic cages and kept in a regulated environment (22 ± 1 °C) with an artificial 12-h light/dark cycle (lighted from 7:00 A.M. to 7:00 P.M.). Food and tap water were available ad libitum. Procedures for pilocarpine-induced status epilepticus (SE) model and all subsequent experiments were approved by the Animal Care and Use Committee at Medical School of Southeast University. All efforts were made to minimize animal suffering and discomfort and to reduce the number of animals used.

### Surgery and virus injection

For adeno-associated viral (AAV) and lentiviral infection, the mice were anesthetized and positioned on a stereotaxic frame (Stoelting, Wood Dale, USA). Vectors (either AAV-Adam10, AAV-Ctrl, or lentiviral shRNA-Adam10, lentiviral shRNA-Ctrl) were bilaterally injected into the hippocampus (coordinates: A/P − 2.2; M/L ± 2.0; D/V 1.9) using 1 μl of viral preparation at a rate of 0.2 μl/min. AAV constructs used were designed and produced by Han Bio (Shanghai, China, contract number: HH20170303RFF-AAV01). Adam10-shRNA lentiviral particles and control lentiviral particles were purchased from Santa Cruz Biotechnology Inc. (Santa Cruz, TX, USA). For EEG recording, the mice were then subjected to hippocampus depth electrode placement as we previously described [18]. A bipolar twist electrode was placed in the left hippocampus (coordinates: A/P − 2.2; M/L − 2.0; D/V 1.9) for continuous EEG monitoring. In addition to the hippocampal electrodes, four cortical screws with two in front of the bregma for bilateral cortex recording and two behind the lambda for ground and reference. Electrodes are connected with a plastic cap and kept in place with dental cement. Animals were allowed to recover for at least 1 week prior to pilocarpine-induced SE.

### Pilocarpine induction of SE and EEG recording

SE model was induced as we previously described [18]. Briefly, the mice were subjected to an intraperitoneal injection of 1 mg/kg methyl-scopolamine (Sigma Aldrich, St. Louis, MO, USA) followed 30 min later by an injection of 300 mg/kg pilocarpine HCL (Sigma Aldrich, St. Louis, MO, USA). Control animals received all drugs and treatments, except they were given saline instead of pilocarpine. After pilocarpine injection, all animals were subjected to continuous video EEG recording with the video EEG monitoring system (Chengyi Inc., Chengdu, China). The seizure intensity was assessed based on Racine scale: stage 1, mouth and facial movements; stage 2, head nodding; stage 3, forelimb clonus; stage 4, seizures characterized by rearing; and stage 5, seizures characterized by rearing and falling [19]. To determine whether neuroinflammation could affect the process of Adam10-regulated epileptogenesis, we treated Adam10 knockdown and control mice with pilocarpine to induce SE, followed by multiple doses of anti-inflammatory agent minocycline (1 mg/kg, Sigma Aldrich, St. Louis, MO, USA) treatment to block neuroinflammation. Animals were then subjected to continuous video EEG recording as described above. Electroencephalographic seizures were differentiated from background noise by the appearance of large-amplitude, high-frequency activity, with the progression of the spike frequency. The behavioral data captured by the synchronized video recording system were used to confirm EEG seizure activity.

### Brain tissue processing

For PCR and Western blot experiments, the hippocampus was dissected, snap-frozen and stored at − 80 °C until use. For immunocytochemistry experiment, the mice were euthanized by an intraperitoneal injection of an overdose of urethane and were transcardially perfused with 100 mL of saline (0.9% *w*/*v* NaCl), followed by 50 mL of 4% paraformaldehyde in 0.05 M sodium phosphate (pH = 7.4, containing 0.8% NaCl). The mouse brains were removed and post-fixed overnight in 4% paraformaldehyde then were cryoprotected in 30% sucrose in PBS for 72 h. The serial coronal hippocampal sections with a thickness of 25 μm were cut using a cryostat (Leica Microsystems, Wetzlar, Germany), and every sixth section throughout the hippocampus was collected in PBS as free-floating sections and was stored at 4 °C for future immunocytochemistry studies as we previously described [20].

### Reverse transcription PCR

The dissected hippocampal tissues were homogenized, and total RNA was extracted with Trizol reagent (Vazyme Biotech, Nanjing, China) according to the manufacturer’s instructions. Total mRNA (1 μg) was reverse transcribed using cDNA RT Kits (Vazyme Biotech, Nanjing, China). RNA and cDNA concentrations were measured using a spectrophotometer (OD-1000, Wuyi Technology, Nanjing, China). For reverse transcription PCR, the reaction conditions were 30 cycles of denaturation at 98 °C for 10 s, annealing at 55 °C for 30 s, and extension at 72 °C for 60 s. PCR products were separated by electrophoresis through a 1.5% agarose gel containing 0.5% μg/ml ethidium bromide and imaged using a Gel imaging system (Tanon, Shanghai, China). The endogenous glyceraldehyde 3-phosphate dehydrogenase (GADPH) gene was used to normalize the level of the target mRNA. The primer sequence of Adam10 and GADPH were as follows: Adam10 forward: 5′-CAACATCAAGGCAAACTATGCGA-3′, reverse: 5′-CTTAGGTTCACTGTCCAAAGCGA-3′; GADPH forward: 5′-AAGGTCATCCCAGAGCTGAAC-3′, reverse: 5′-TGAAGTCGCAGGAGACAACC-3′.

### Western blotting

The dissected hippocampal tissues of the mice were homogenized in tissue lysis buffer (Beyotime Biotech, China). After being lysed for 15 min on ice, the samples were centrifuged at 12,000 rpm for 15 min. The protein content in each supernatant fraction was determined using a BCA protein assay kit (Pierce, Rockford, IL, USA), and samples containing equivalent amounts of protein were applied to 12% acrylamide denaturing gels (SDS-PAGE). After electrophoresis, the proteins were transferred to nitrocellulose membranes (Amersham, Little Chalfont, UK) using a Bio-Rad mini-protein-III wet transfer unit (Hercules, CA, USA) overnight at 4 °C. The membranes were then incubated with 5% non-fat milk in TBST (10 mmol/l Tris pH = 7.6, 150 mmol/L NaCl, 0.01%Tween-20) for 1 h at room temperature followed by three washes then were incubated with mouse anti-Adam10 (1:2000; Santa Cruz, TX, USA), rabbit anti-iNOS (1:5000; Abcam, Temecula, CA, USA), rabbit anti-COX-2 (1:2500; Abcam, Temecula, CA, USA), mouse anti-NF-κB (1:2500; Santa Cruz, TX, USA), and rabbit anti-β-actin (1:5000; Sigma-Aldrich, St. Louis, USA) in TBST overnight at 4 °C. After several washes with TBST buffer, the membranes were incubated for 1 h with HRP-linked secondary antibody (Boster Bioengineering, Wuhan, China) diluted 1:5,000, followed by four washes. The membranes were then processed with enhanced chemiluminescence (ECL) Western blot detection reagents (Millipore, Billerica, MA, USA). Signals were digitally captured using a MicroChemi chemiluminescent image analysis system (DNR Bio-imaging Systems, Jerusalem, Israel). Blots were quantified using the ImageJ software (NIH, Bethesda, MD, USA).

### Immunocytochemistry

The immunocytochemistry studies were performed on free-floating sections as described previously [20]. Briefly, the sections were heated (65 °C for 50 min) in antigen unmasking solution (2xSSC/formamide), incubated in 2 M HCl (30 °C for 30 min), rinsed in 0.1 M boric acid (pH 8.5) for 10 min, incubated in 1% H_2_O_2_ in PBS for 30 min, and blocked in PBS containing 3% normal goat serum, 0.3% (*w*/*v*) Triton X-100, and 0.1% BSA (room temperature for 1 h), followed by incubation with mouse anti-Adam10 (1:200; Santa Cruz, TX, USA), rabbit anti-Iba-1 (1:200; Wako, Osaka, Japan), and mouse anti-GFAP (1:100, Boster, Bioengineering, Wuhan, China) antibody at 4 °C overnight. For DAB staining, the sections were developed with super ABC kit (Boster, Wuhan, China). For immunofluorescence assay, the sections were incubated with a TRITC-conjugated goat anti-rabbit antibody (1:200; Cwbiotech, Beijing, China) for Iba-1 staining and a TRITC-conjugated goat anti-mouse antibody (1:200; Cwbiotech, Beijing, China) for Adam10 and GFAP staining, respectively. The sections were then rinsed and mounted on gelatin-coated slides in DAPI antifade mounting medium (SouthernBiotech, Birmingham, AL, USA). The images of Adam10, Iba-1, and GFAP staining were captured with a confocal laser scanning microscope (Olympus LSM-GB200, Japan). The quantitative analyses of the Adam10, Iba-1, and GFAP immunostaining were performed using the ImageJ software (NIH, Bethesda, MD, USA) as described in our previous study [21, 22].

### Enzyme-linked immunosorbent assay

The mouse IL-1β and TNF-α ELISA was performed according to the manufacturer’s protocol. Briefly, hippocampal lysates were incubated with reaction buffer. The mixture was incubated for 2.5 h at room temperature before protease activity was detected using a microplate reader (BioTek, USA). The samples for each ELISA were run in duplicate, and each ELISA was repeated at least three times, using the mouse IL-1β and TNF-α ELISA kits (ExCell Bio, Shanghai, China).

### Statistical analysis

All data are presented as the means ± SEM. Statistical significance was determined by using unpaired two-tailed Student’s *t* test for the two groups’ comparison and by using one-way or two-way ANOVA for multi-group comparisons. Tukey’s test was used for post hoc comparisons. Differences were considered to be significant for values of *p* < 0.05.

## Results

### Adam10 expression is decreased in the hippocampus of pilocarpine-induced SE mice

**Fig. 1:**
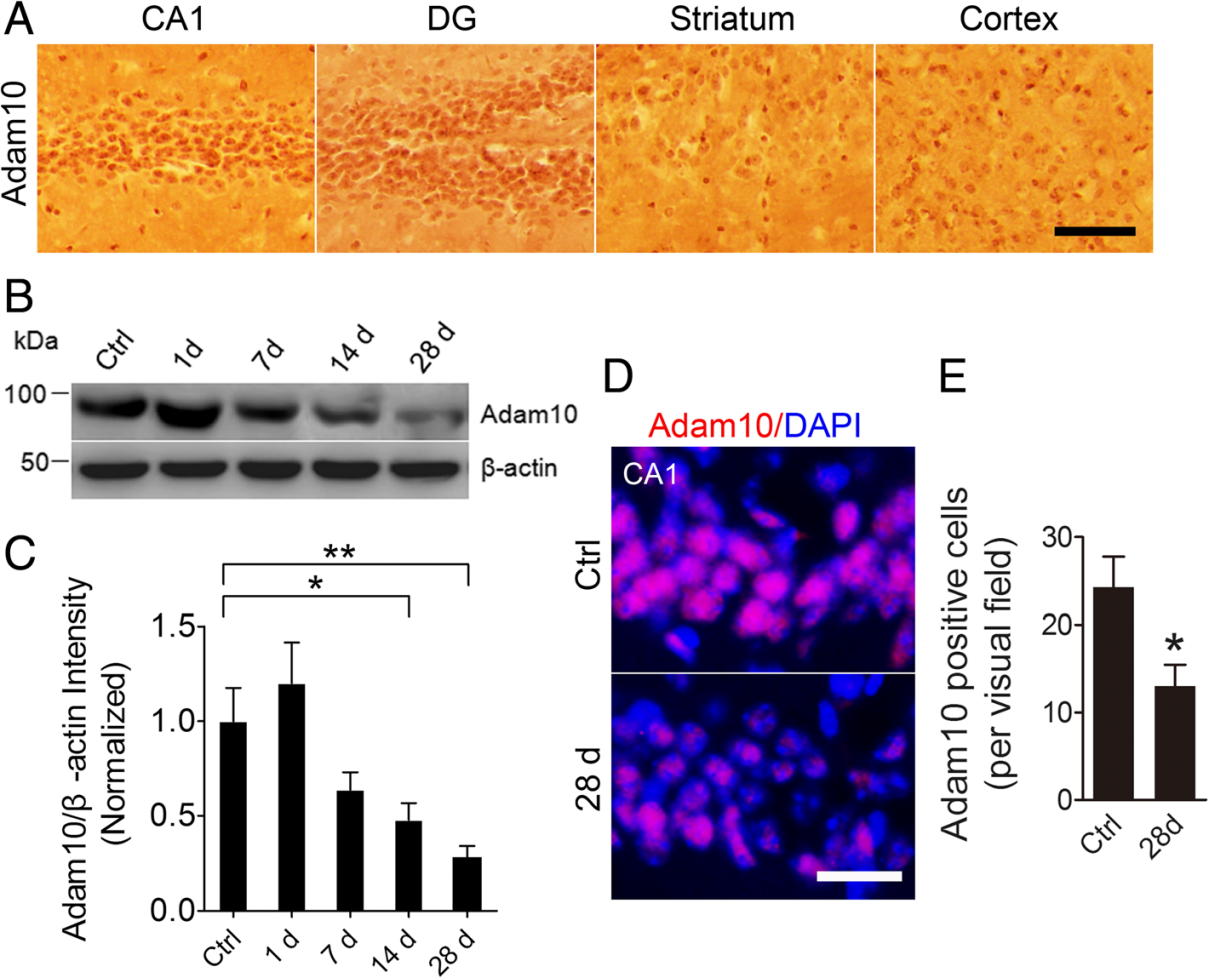
Adam10 expression is decreased in the hippocampus of TLE mice. **a** Representative images showing the expression of Adam10 protein in the CA1, DG, striatum, and cortex area of the mouse brain by DAB staining (*n* = 4). **b**, **c** Western blots and quantification of Adam10 protein level in Ctrl and days 1, 7, 14, and 28 post-SE mice (*F*_4,20_ = 7.40, *p* = 0.020, 14 days vs Ctrl; *p* = 0.006, 28 days vs Ctrl) (*n* = 5). **d** Representative images of the immunostaining of Adam10 in the hippocampal CA1 region of the Ctrl and day 28 post-SE mice, respectively. **e** Bar graphs showing the quantification of Adam10-positive cells in Ctrl and day 28 post-SE mice (*p* = 0.02) (*n* = 4). *p < 0.05 and **p < 0.01 compared with Ctrl mice, unpaired two-tailed Student’s *t* test, and one-way ANOVA. Scale bar = 100 μm in **a** and 20 μm in **d**

A growing body of evidence suggests a possible link between Adam10 and epilepsy [8, 10, 23, 24]. To test this hypothesis, we first assessed Adam10 expression in different brain regions of mice. Our immunohistochemistry results show clear nuclear staining of Adam10 in the hippocampal CA1 region, DG, striatum, and cortex, with strong Adam10 expression in the hippocampal CA1 region and DG (Fig. 1a), suggesting that Adam10 may have important functions in the hippocampus. A great number of clinical and experimental studies have consistently reported that the hippocampus is involved in the generation and propagation of seizures in the brain [25–30]. Based on these facts, we speculate that Adam10 plays an important role in the development of epilepsy through regulation of neural activities in the hippocampus.

To investigate the expression pattern of Adam10 in the hippocampus of TLE mice, we examined the hippocampal Adam10 protein levels following pilocarpine-induced SE, which serves as a model of TLE. Our Western blotting data show that Adam10 protein levels in the hippocampus start to progressively decrease from day 14 to day 28 post-SE (Fig. 1b, c). Immunofluorescence data reveal that Adam10-positive cells in the hippocampal CA1 region are significantly decreased at day 28 post-SE compared to those of the control animals (Fig. 1d, e), which further confirmed the decrease of Adam10 expression in the hippocampus of pilocarpine-induced SE mice. Taken together, these results indicate that pilocarpine-induced SE results in a progressive decrease of Adam10 expression in a time-dependent manner.

### Neuroinflammation is triggered in the hippocampus after pilocarpine-induced SE

**Fig. 2:**
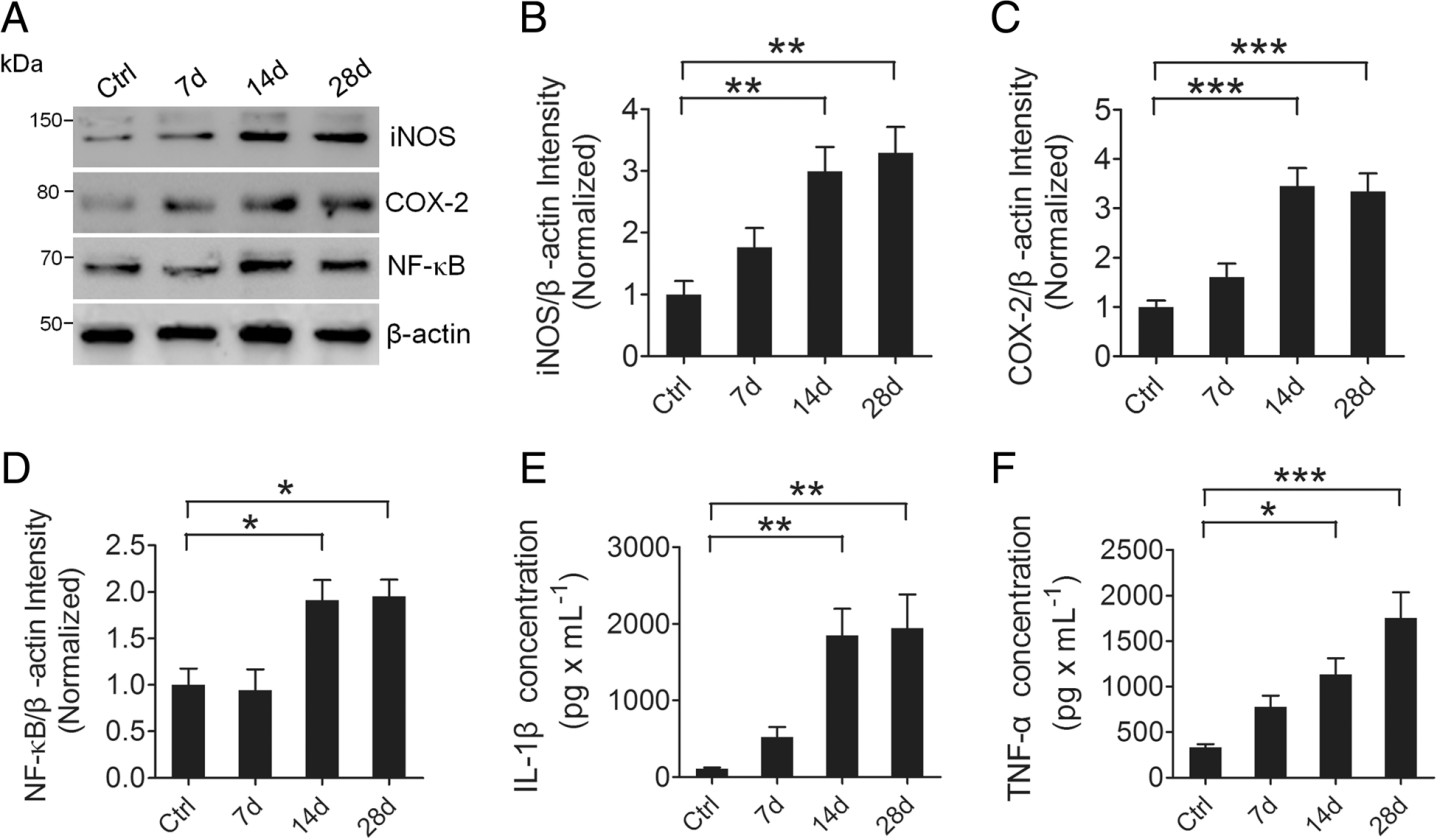
Inflammation-related proteins and cytokines are increased in the hippocampus of TLE mice. **a** Western blotting showing the protein levels of the inflammation-related proteins iNOS and COX-2 and NF-κB in the hippocampus of Ctrl and 7, 14, and 28 days post-SE mice. **b**–**d** Bar graphs showing the quantification of iNOS (*F*_3,16_ = 9.60, *p* = 0.004, 14 days vs Ctrl; *p* = 0.001, 28 days vs Ctrl), COX-2 (*F*_3,16_ = 17.02, *p* < 0.001, 14 days vs Ctrl; *p* < 0.001, 28 days vs Ctrl), and NF-κB (*F*_3,16_ = 7.76, *p* = 0.025, 14 days vs Ctrl; *p* = 0.018, 28 days vs Ctrl), which are represented as the intensity ratios of these proteins to β-actin (*n* = 5). **e**, **f** Bar graphs showing the concentration of IL-1β (*F*_3,16_ = 10.32, *p* = 0.004, 14 days vs Ctrl; *p* = 0.002, 28 days vs Ctrl) and TNF-α (*F*_3, 16_ = 11.41, *p* = 0.027, 14 days vs Ctrl; *p* < 0.001, 28 days vs Ctrl) in the hippocampus of Ctrl and 7, 14, and 28 day post-SE mice, which were detected by ELISA (*n* = 5). *p < 0.05, **p < 0.01, ***p < 0.001, and one-way ANOVA

Neuroinflammation is implicated as a pathogenic mechanism in a variety of neurological disorders including epilepsy. To determine whether neuroinflammation is present in the hippocampus of pilocarpine-induced SE mice, we first examined the inflammatory mediators iNOS and COX-2 and the transcription factor NF-κB, which is responsible for the induction of inflammatory mediators in the hippocampus of pilocarpine-induced SE mice. Our Western blotting results reveal that the hippocampal protein levels of the inflammatory mediators iNOS and COX-2 and the transcription factor NF-κB are significantly increased at days 14 and 28 post-SE compared to those of the control animals (Fig. 2a–d). To further confirm the neuroinflammation in the hippocampus of SE mice, we detected the levels of the cytokines IL-1β and TNF-α by ELISA. Notably, we find that, similar to the changing trend of the above inflammatory mediators and the transcription factor in the hippocampus of SE mice, the production of IL-1β and TNF-α is significantly increased at days 14 and 28 post-SE compared to those of the control mice (Fig. 2e, f). Taken together, these results suggest that SE triggers neuroinflammation in the hippocampus.

### Overexpression of Adam10 decreases spontaneous seizures in TLE mice

**Fig. 3:**
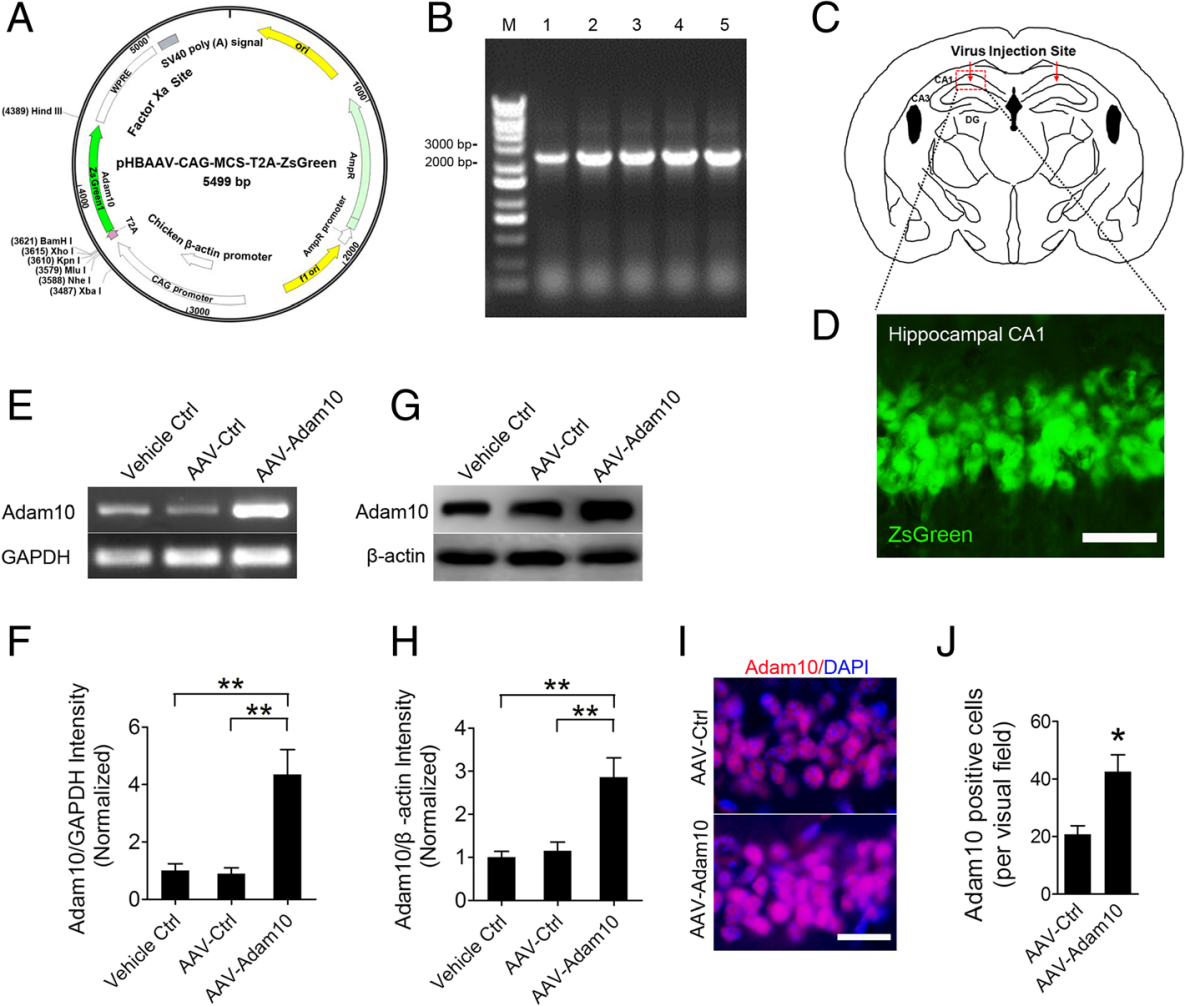
AAV-Adam10 vector construction and hippocampal Adam10 overexpression verification. **a** Structure of pHBAAV-CAG-MCS-T2A-ZsGreen AAV vector, which carries Adam10 and an empty construct or a ZsGreen gene as an indicator. **b** Single-clone PCR identification of Adam10 expression (lanes 1–5). **c** Graphic illustration of the AAV bilateral injection sites in the hippocampus of the mouse brain. Arrows indicate the bilateral injection sites in the hippocampal CA1 region. **d** Distribution of AAV-mediated ZsGreen expression in the CA1 region of the hippocampus. **e**, **f** RT-PCR analysis of Adam10 expression in the hippocampus of Vehicle Ctrl, AAV-Ctrl, and AAV-Adam10 mice, respectively (*F*_2,12_ = 13.41, *p* = 0.007, AAV-Adam10 vs Vehicle Ctrl; *p* = 0.004, AAV-Adam10 vs AAV-Ctrl) (*n* = 5). **g**, **h** Western blot analysis of Adam10 protein levels in the hippocampus of Vehicle Ctrl, AAV-Ctrl, and AAV-Adam10 mice, respectively (*F*_2,12_ = 12.06, *p* = 0.002, AAV-Adam10 vs Vehicle Ctrl; *p* = 0.004, AAV-Adam10 vs AAV-Ctrl) (*n* = 5). **i** Representative images of the Adam10 immunostaining in the hippocampal CA1 region of AAV-Ctrl and AAV-Adam10 mice, respectively. **j** Bar graphs showing the quantification of Adam10-positive cells in the hippocampal CA1 region of the AAV-Ctrl and AAV-Adam10 mice, respectively (*p* = 0.012) (*n* = 4). *p < 0.05, **p < 0.01, unpaired two-tailed Student’s *t* test, and one-way ANOVA. Scale bar = 50 μm in **d** and 20 μm in **i**

The AAV vector carrying Adam10 and an empty construct or a ZsGreen gene, which is a green fluorescent protein to be used as an indicator (Fig. 3a). Single-clone PCR identification of Adam10 expression is shown in Fig. 3b. Each AAV vector was bilaterally injected into the hippocampal CA1 region (Fig. 3c). As expected, in the AAV-ZsGreen-treated mice, hippocampal CA1 neurons show robust expression of ZsGreen 2 weeks after the virus injection (Fig. 3d), suggesting high AAV infection efficiency. Quantification of mRNA (Fig. 3e, f) and protein (Fig. 3g, h) levels by RT-PCR and Western blotting 2 weeks after the virus injection validated the overexpression of Adam10 in the mouse hippocampus. Further analysis of Adam10 expression in the hippocampal CA1 region by immunofluorescence reveals that Adam10-overexpressing mice show a higher percentage of Adam10-positive cells than those of the control mice (Fig. 3i, j), which further confirmed the overexpression of Adam10 in the hippocampus.

**Fig. 4:**
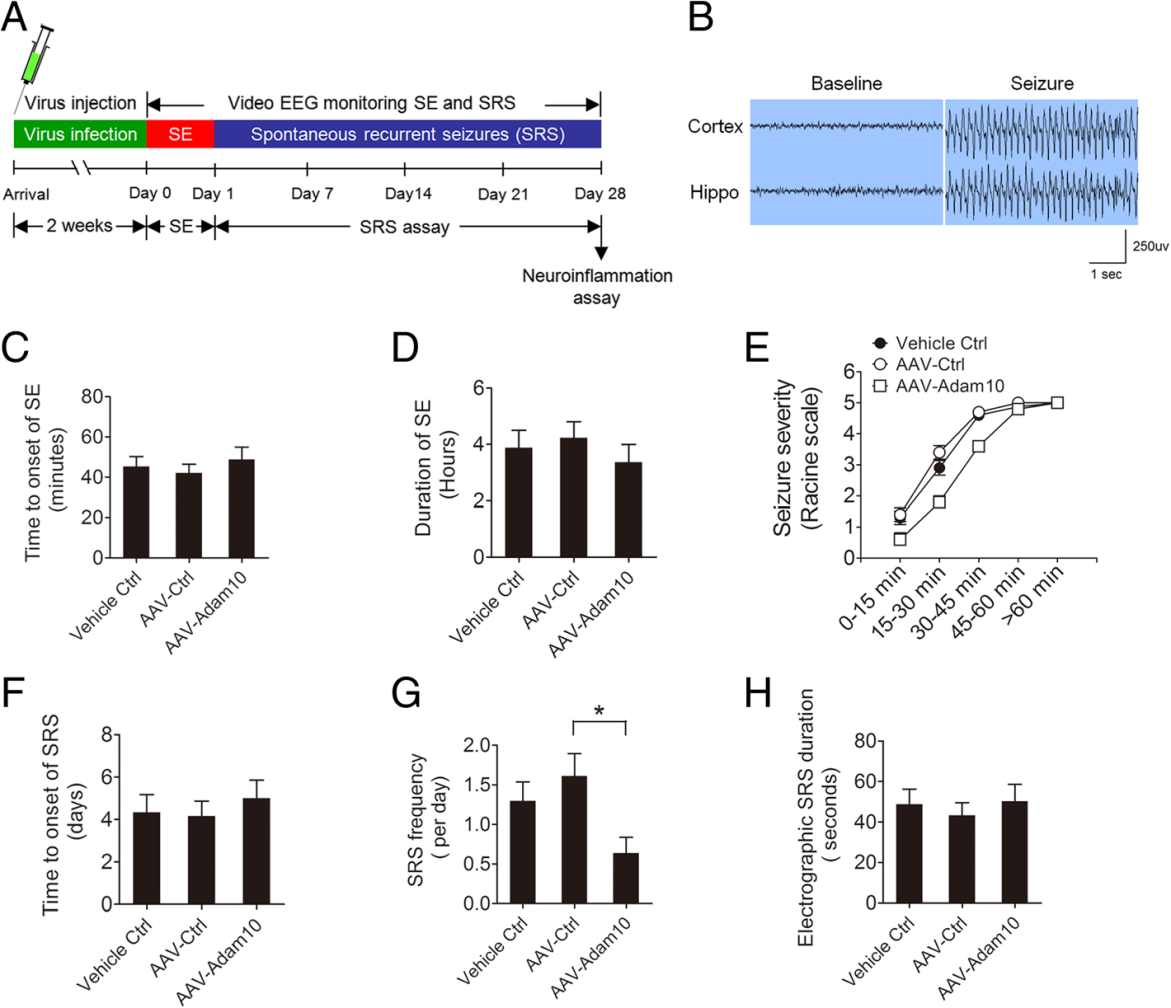
Adam10 overexpression decreases spontaneous seizures in TLE mice. **a** Schematic diagram of the experimental design. Mice were bilaterally injected with the virus into the hippocampus, and after 2 weeks of recovery, these mice were induced for SE and continuously video EEG monitored for 4 weeks for SE and SRS analysis. These mice were then sacrificed after the EEG recording was completed at day 28 post-SE to detect hippocampal neuroinflammation. **b** A typical EEG recording of the baseline and seizure in the cortex and hippocampus. **c** Bar graph showing the average time to onset of SE in the Vehicle Ctrl, AAV-Ctrl, and AAV-Adam10 mice (*n* = 12). **d** Bar graph showing the quantification of SE duration in the Vehicle Ctrl, AAV-Ctrl, and AAV-Adam10 mice (*n* = 10). **e** Line graphs showing the seizure severity during SE development in the Vehicle Ctrl, AAV-Ctrl, and AAV-Adam10 mice (at 15–30 min, *F*_2,27_ = 14.02, *p* = 0.004, AAV-Adam10 vs Vehicle Ctrl; *p* < 0.001, AAV-Adam10 vs AAV-Ctrl; at 30–45 min, *F*_2,27_ = 7.41, *p* = 0.032, AAV-Adam10 vs Vehicle Ctrl; *p* = 0.026, AAV-Adam10 vs AAV-Ctrl) (*n* = 10). **f** Bar graph showing the average time to onset of first spontaneous seizure in the Vehicle Ctrl, AAV-Ctrl, and AAV-Adam10 mice (*n* = 6). **g** Bar graph showing the SRS frequency in the Vehicle Ctrl, AAV-Ctrl, and AAV-Adam10 mice (*F*_2,15_ = 4.23, *p* = 0.031, AAV-Adam10 vs AAV-Ctrl) (*n* = 6). **h** Bar graph showing the quantification of electrographic SRS duration in the Vehicle Ctrl, AAV-Ctrl, and AAV-Adam10 mice (*n* = 6). *p < 0.05 and one-way ANOVA

To determine whether the overexpression of Adam10 affects the epileptogenesis in the pilocarpine-induced TLE mice, we bilaterally treated the hippocampus of the mice with Vehicle Ctrl, AAV-Ctrl, or AAV-Adam10, followed by pilocarpine-induced SE. All animals were subjected to continuous video EEG monitoring from the start of SE induction until 4 weeks following SE (Fig. 4a). EEG recording shows the burst of large amplitude and high-frequency spikes in both the cortex and hippocampus of SE mice (Fig. 4b). SE analysis shows that AAV-Adam10 treatment did not alter the onset of SE (Fig. 4c) as well as SE duration (Fig. 4d). AAV-Adam10 treatment reduced seizure severity at 15–30 and 30–45 min after SE (Fig. 4e). Following the episode of SE, we monitored the spontaneous recurrent seizures (SRS) by video EEG recording continuously for 4 weeks. Our data reveal that the latency to the onset of SRS and the electrographic SRS duration remain similar between the AAV-Adam10-treated and the control mice (Fig. 4f, h). However, AAV-Adam10 treatment significantly decreased SRS frequency (Fig. 4g). Taken together, these results suggest that overexpression of Adam10 in the hippocampus decreases spontaneous seizures in TLE mice.

### Overexpression of Adam10 suppresses SE-induced hippocampal neuroinflammation

**Fig. 5:**
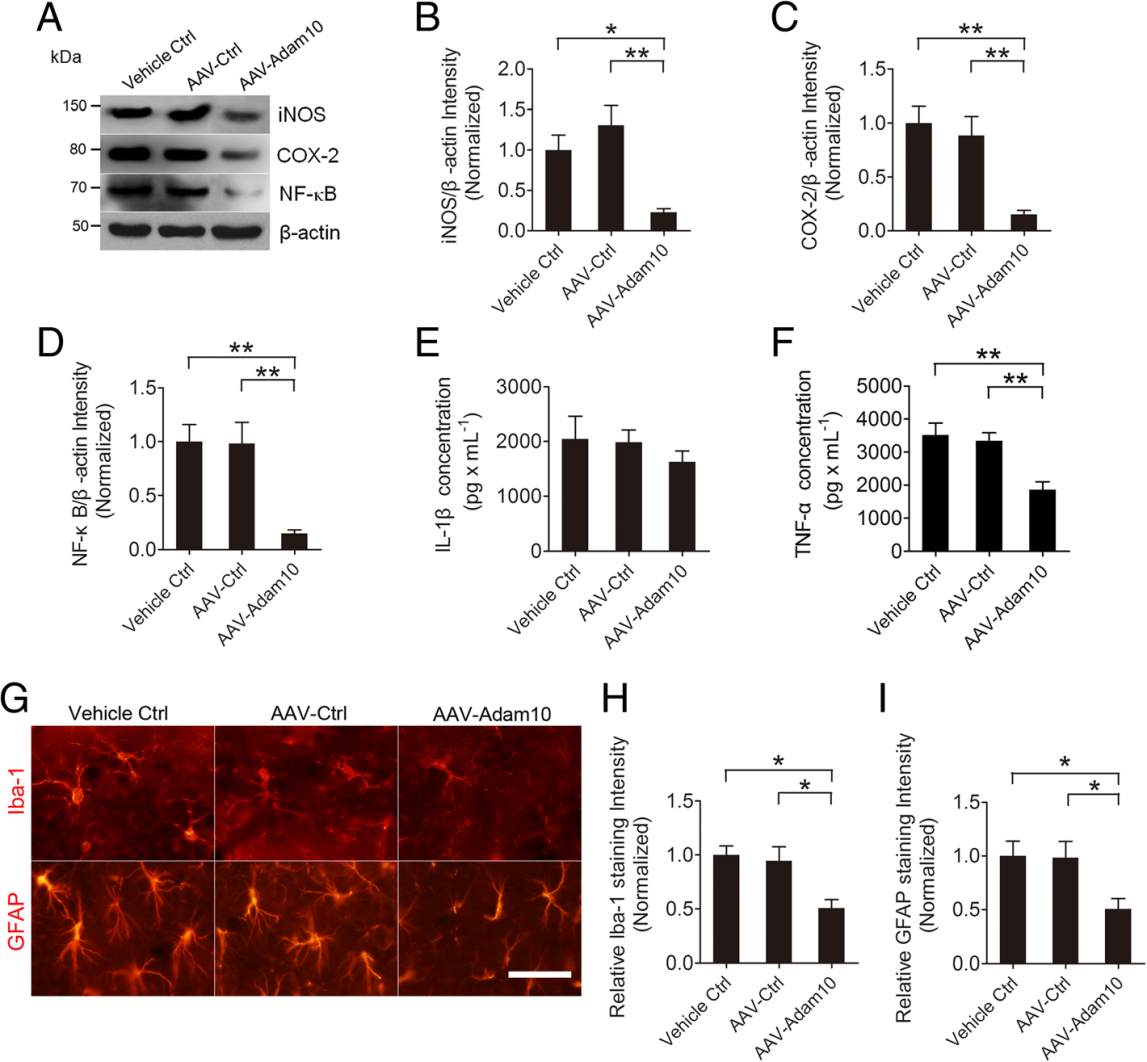
Adam10 overexpression suppresses hippocampal neuroinflammation in TLE mice. **a** Western blotting showing the protein levels of inflammation-related proteins iNOS and COX-2 and NF-κB in the hippocampus of Vehicle Ctrl, AAV-Ctrl, and AAV-Adam10-treated TLE mice. **b**–**d** Bar graphs showing the quantification of iNOS (*F*_2,12_ = 9.86, *p* = 0.024, AAV-Adam10 vs Vehicle Ctrl; *p* = 0.003, AAV-Adam10 vs AAV-Ctrl), COX-2 (*F*_2,12_ = 11.27, *p* = 0.003, AAV-Adam10 vs Vehicle Ctrl; *p* = 0.007, AAV-Adam10 vs AAV-Ctrl), and NF-κB (*F*_2,12_ = 11.05, *p* = 0.004, AAV-Adam10 vs Vehicle Ctrl; *p* = 0.005, AAV-Adam10 vs AAV-Ctrl), which were represented as the intensity ratios of these proteins to β-actin (*n* = 5). **e**, **f** Bar graphs showing the concentration of IL-1β (*F*_2,12_ = 0.59, *p* = 0.572) and TNF-α (*F*_2,12_ = 10.11, *p* = 0.004, AAV-Adam10 vs Vehicle Ctrl; *p* = 0.009, AAV-Adam10 vs AAV-Ctrl) in the hippocampus of Ctrl, AAV-Ctrl, and AAV-Adam10-treated TLE mice as detected by ELISA (*n* = 5). **g** Representative images of the immunostaining of Iba-1 and GFAP in the hippocampal CA1 region of the Ctrl, AAV-Ctrl, and AAV-Adam10 mice, respectively. **h**, **i** Bar graphs showing the quantification of Iba-1- (*F*_2,12_ = 7.31, *p* = 0.012, AAV-Adam10 vs Vehicle Ctrl; *p* = 0.024, AAV-Adam10 vs AAV-Ctrl) and GFAP (*F*_2,12_ = 4.61, *p* = 0.039, AAV-Adam10 vs Vehicle Ctrl; *p* = 0.042, AAV-Adam10 vs AAV-Ctrl)-positive cells in the hippocampal CA1 region of the Ctrl, AAV-Ctrl, and AAV-Adam10 mice, respectively (*n* = 4). *p < 0.05, **p < 0.01, and one-way ANOVA. Scale bar = 50 μm in **g**

A recent study indicates that Adam10 is involved in the process of neuroinflammation [16]. To investigate whether Adam10 regulates the neuroinflammation in the hippocampus of TLE mice, we treated mice with AAV-Adam10 in order to overexpress Adam10 in the hippocampus, followed by pilocarpine-induced SE. Four weeks after SE, we examined the hippocampal neuroinflammation (Fig. 4a). Our Western blotting results reveal that the inflammatory mediators iNOS and COX-2 and the inflammatory transcription factor NF-κB are significantly suppressed by hippocampal Adam10 overexpression (Fig. 5a–d). ELISA reveals that after hippocampal Adam10 overexpression, IL-β production is slightly reduced (Fig. 5e), while TNF-α production is significantly decreased compared to those levels in the control animals (Fig. 5f). We next investigated the effects of Adam10 overexpression on glial activation in the hippocampus of TLE mice. Our immunofluorescence data reveal that 4 weeks after SE, the fluorescence intensities of both Iba-1 and GFAP are decreased in the hippocampus of Adam10-overexpressing mice compared to those levels in the control mice (Fig. 5g–i). Notably, the image analysis shows that, in comparison with the control mice, the numbers of GFAP and Iba-1 immunopositive cells in the CA1 region of AAV-Adam10-treated mice are dramatically reduced, and the cells are forming fewer ramifications (Fig. 5g). Taken together, these data suggest that the overexpression of Adam10 suppresses SE-induced hippocampal neuroinflammation.

### Knockdown of Adam10 increases spontaneous seizures in TLE mice

**Fig. 6:**
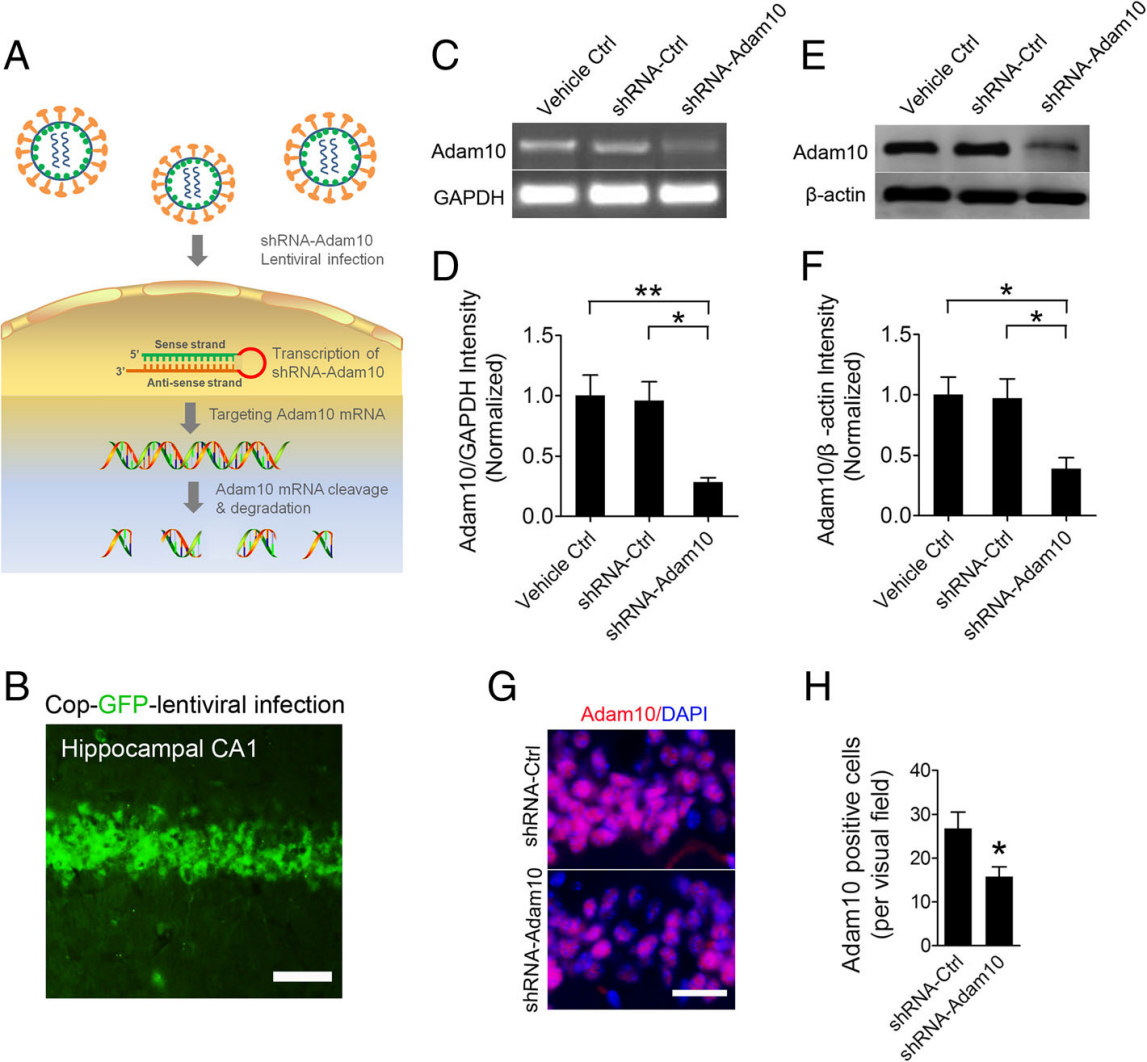
shRNA-mediated Adam10 knockdown in the hippocampus. **a** Action mode of shRNA-mediated Adam10 knockdown via lentiviral vector infection. **b** Distribution of lentivirus-mediated cop-GFP expression in the CA1 region of the hippocampus. **c**, **d** RT-PCR analysis of Adam10 expression in the hippocampus of Vehicle Ctrl, shRNA-Ctrl, and shRNA-Adam10 mice, respectively (*F*_2,12_ = 8.84, *p* = 0.008, shRNA-Adam10 vs Vehicle Ctrl; *p* = 0.032, shRNA-Adam10 vs shRNA-Ctrl) (*n* = 5). **e**, **f** Western blot analysis of Adam10 protein levels in the hippocampus of Vehicle Ctrl, shRNA-Ctrl, and shRNA-Adam10 mice, respectively (*F*_2,12_ = 6.38, *p* = 0.021, shRNA-Adam10 vs Vehicle Ctrl; *p* = 0.027, shRNA-Adam10 vs shRNA-Ctrl) (*n* = 5). **g** Representative images of the immunostaining of Adam10 in the hippocampal CA1 region of the shRNA-Ctrl and shRNA-Adam10 mice, respectively. **h** Bar graph showing the quantification of Adam10-positive cells in the hippocampal CA1 region of shRNA-Ctrl and shRNA-Adam10 mice, respectively (*p* = 0.014) (*n* = 4). *p < 0.05, **p < 0.01, unpaired two-tailed Student’s *t* test, and one-way ANOVA. Scale bar = 50 μm in **b** and 20 μm in **g**

To investigate whether reducing Adam10 expression could play a role in the epileptogenesis in TLE mice, we bilaterally injected lentivirus carrying control or Adam10-shRNA (Fig. 6a) into the hippocampal CA1 regions of mice. A cop-GFP control lentiviral particle, which contains the full-length cop-GFP gene for high-level expression of the fluorescent protein, was used to test the lentiviral infection efficiency. Our results show a robust cop-GFP expression in the hippocampal CA1 region (Fig. 6b), suggesting high lentiviral infection efficiency. Furthermore, the efficiency of Adam10 silencing was confirmed by RT-PCR and Western blotting analysis 2 weeks after the lentivirus injection, which respectively show a significant reduction of mRNA (Fig. 6c, d) and protein (Fig. 6e, f) levels of Adam10. Immunofluorescence reveals that shRNA-Adam10-treated mice showed fewer Adam10-positive cells than those of the shRNA-control mice (Fig. 6g, h), which confirmed the knockdown of Adam10 in the hippocampus.

**Fig. 7:**
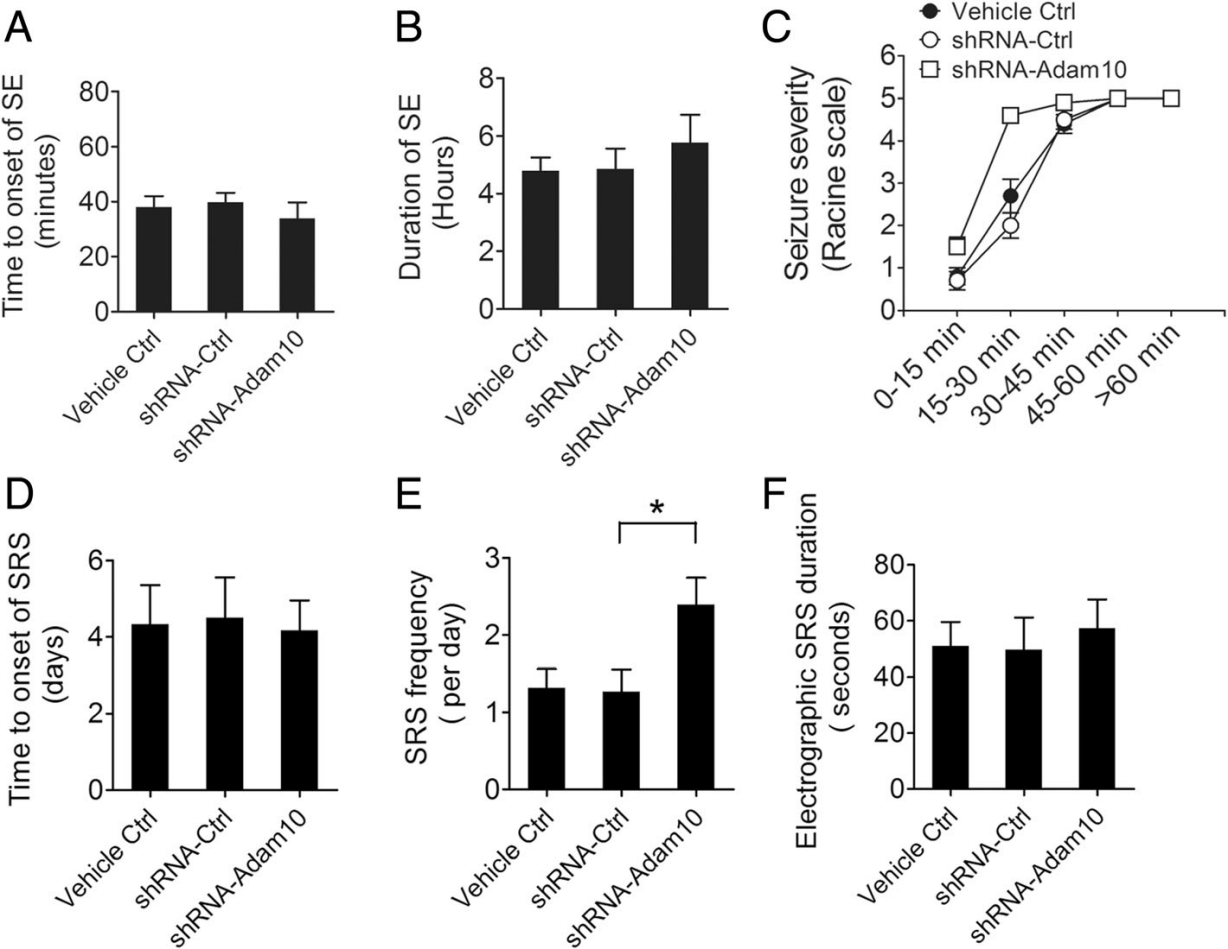
Adam10 knockdown increases spontaneous seizures in TLE mice. **a** Bar graph showing the average time to onset of SE in the Vehicle Ctrl, shRNA-Ctrl, and shRNA-Adam10 mice (*n* = 12). **b** Bar graph showing the quantification of SE duration in the Vehicle Ctrl, shRNA-Ctrl, and shRNA-Adam10 mice (*n* = 10). **c** Line graphs showing the seizure severity during SE development in the Vehicle Ctrl, shRNA-Ctrl, and shRNA-Adam10 mice (at 15–30 min, *F*_2,27_ = 19.95, *p* < 0.001, shRNA-Adam10 vs Vehicle Ctrl; *p* < 0.001, shRNA-Adam10 vs shRNA-Ctrl) (*n* = 10). **d** Bar graph showing the average time to onset of the first spontaneous seizure in the Vehicle Ctrl, shRNA-Ctrl, and shRNA-Adam10 mice (*n* = 6). **e** Bar graph showing the SRS frequency in the Vehicle Ctrl, shRNA-Ctrl, and shRNA-Adam10 mice (*F*_2,15_ = 4.55, *p* = 0.043, shRNA-Adam10 vs shRNA-Ctrl) (*n* = 6). **f** Bar graph showing the quantification of electrographic SRS duration in the Vehicle Ctrl, shRNA-Ctrl, and shRNA-Adam10 mice (*n* = 6). *p < 0.05 and one-way ANOVA

To further determine whether Adam10 affects epileptogenesis in the pilocarpine-induced SE mice, we treated mice with Vehicle Ctrl, lentivirus carrying shRNA-Ctrl, and shRNA-Adam10 in the hippocampus, followed by pilocarpine-induced SE. All animals were subjected to continuous video EEG monitoring as described in Fig. 4a. We then analyzed the SE episode and SRS of Vehicle Ctrl and shRNA-Ctrl- and shRNA-Adam10-treated mice. SE analysis shows that shRNA-Adam10 treatment did not alter the onset of SE (Fig. 7a) as well as SE duration (Fig. 7b). Furthermore, shRNA-Adam10 treatment increased the seizure severity at 15–30 min after SE (Fig. 7c). Following the episode of SE, we monitored the SRS continuously for 4 weeks. Our data show that the latency to the onset of SRS and the electrographic SRS duration remain similar between the shRNA-Adam10-treated and the control mice (Fig. 7d, f). However, shRNA-Adam10 treatment significantly increased the SRS frequency (Fig. 7e). Taken together, these results suggest that knockdown of Adam10 increases spontaneous seizures in pilocarpine-induced TLE mice.

### Knockdown of Adam10 exacerbates hippocampal neuroinflammation in TLE mice

**Fig. 8:**
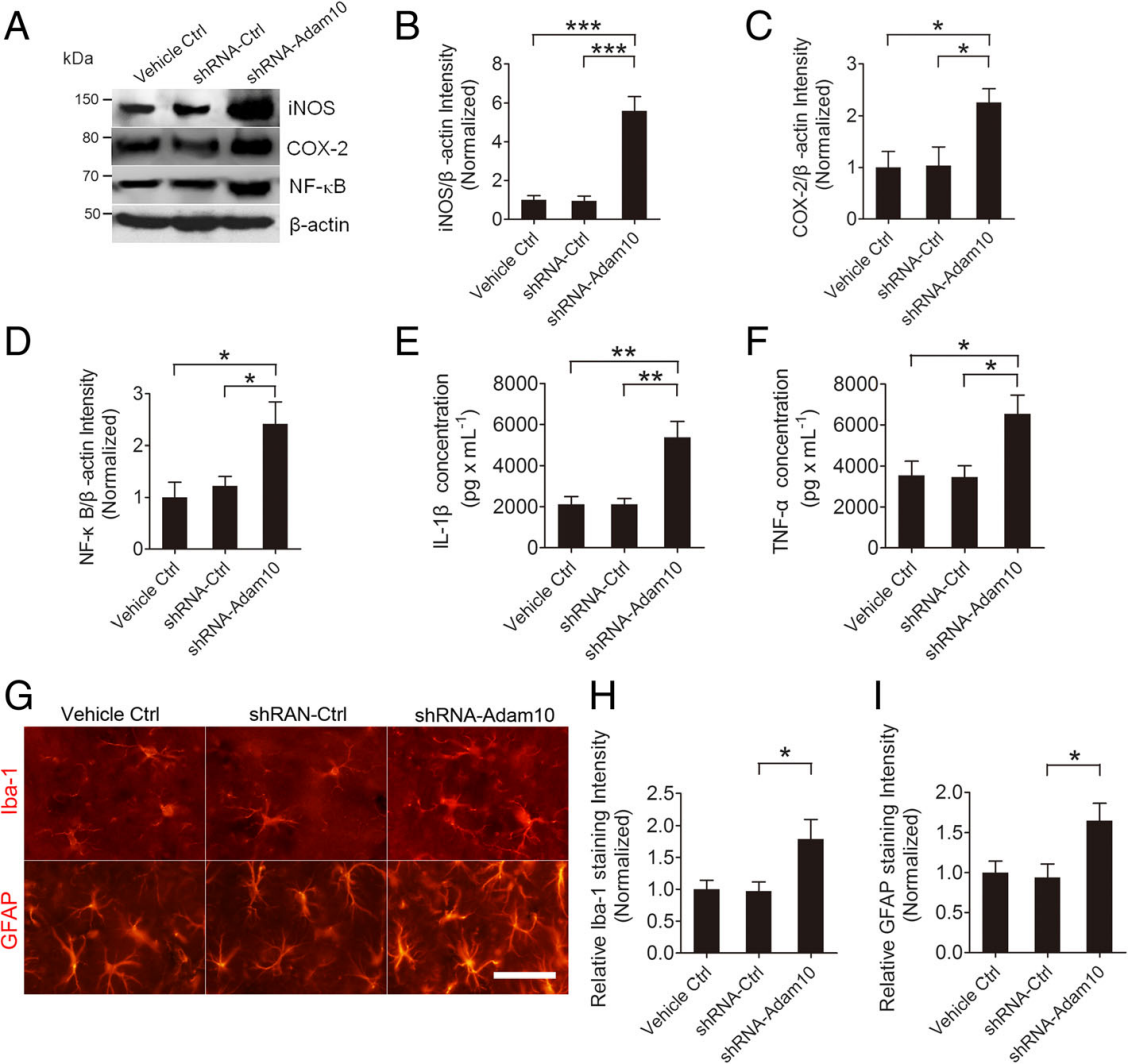
Adam10 knockdown exacerbates hippocampal neuroinflammation in TLE mice. **a** Western blotting showing the protein levels of the inflammation-related proteins iNOS and COX-2 and NF-κB in the hippocampus of Vehicle Ctrl, shRNA-Ctrl, and shRNA-Adam10-treated TLE mice. **b**–**d** Bar graphs showing the quantification of iNOS (*F*_2,12_ = 32.09, *p* < 0.001, shRNA-Adam10 vs Vehicle Ctrl; *p* < 0.001, shRNA-Adam10 vs shRNA-Ctrl), COX-2 (*F*_2,12_ = 5.32, *p* = 0.035, shRNA-Adam10 vs Vehicle Ctrl; *p* = 0.041, shRNA-Adam10 vs shRNA-Ctrl), and NF-κB (*F*_2,12_ = 5.82, *p* = 0.020, shRNA-Adam10 vs Vehicle Ctrl; *p* = 0.049, shRNA-Adam10 vs shRNA-Ctrl), which were represented as the intensity ratios of these proteins to β-actin (*n* = 5). **e**, **f** Bar graphs showing the concentration of IL-1β (*F*_2,12_ = 12.78, *p* = 0.003, shRNA-Adam10 vs Vehicle Ctrl; *p* = 0.003, shRNA-Adam10 vs shRNA-Ctrl) and TNF-α (*F*_2,12_ = 5.66, *p* = 0.035, shRNA-Adam10 vs Vehicle Ctrl; *p* = 0.030, shRNA-Adam10 vs shRNA-Ctrl) in the hippocampus of Vehicle Ctrl, shRNA-Ctrl, and shRNA-Adam10-treated TLE mice, as detected by ELISA (*n* = 5). **g** Representative images of the immunostaining of Iba-1 and GFAP in the hippocampal CA1 region of the Vehicle Ctrl, shRNA-Ctrl, and shRNA-Adam10 mice, respectively. **h**, **i** Bar graphs showing the quantification of Iba-1- (*F*_2,12_ = 4.70, *p* = 0.047, shRNA-Adam10 vs shRNA-Ctrl) and GFAP (*F*_2,12_ = 4.88, *p* = 0.039, shRNA-Adam10 vs shRNA-Ctrl)-positive cells in the hippocampal CA1 region of the Vehicle Ctrl, shRNA-Ctrl, and shRNA-Adam10 mice, respectively (*n* = 4). *p < 0.05, **p < 0.01, ***p < 0.001, and one-way ANOVA. Scale bar = 50 μm in **g**

We next determined whether the knockdown of Adam10 had any effect on hippocampal neuroinflammation in pilocarpine-induced SE mice. For this purpose, we first examined the inflammatory mediators iNOS and COX-2 and the inflammatory transcription factor NF-κB by Western blotting. Our results reveal that both the inflammatory mediators iNOS and COX-2 and the inflammatory transcription factor NF-κB are significantly increased in shRNA-Adam10-treated mice compared to the levels in Vehicle Ctrl- and shRNA-Ctrl-treated mice 4 weeks after SE (Fig. 8a–d). Consistently, ELISA reveals that after the hippocampal Adam10 knockdown, both IL-β and TNF-α productions are significantly increased (Fig. 8e, f). We next investigated the effects of Adam10 knockdown on glial activation in the hippocampus. Our immunofluorescence data reveal that 4 weeks after SE, the fluorescence intensities of both Iba-1 and GFAP are significantly increased in the hippocampus of Adam10 knockdown mice compared to those of the control mice (Fig. 8g–i). Notably, the image analysis shows that the numbers of GFAP and Iba-1 immunopositive cells in the CA1 region of shRNA-Adam10-treated mice are dramatically increased in comparison with those of Vehicle Ctrl and shRNA-Ctrl mice, and the cells form more ramifications (Fig. 8g). Taken together, these data suggest that knockdown of Adam10 exacerbates hippocampal neuroinflammation in pilocarpine-induced TLE mice.

### Increased seizure activity by Adam10 knockdown is dependent on hippocampal neuroinflammation

**Fig. 9:**
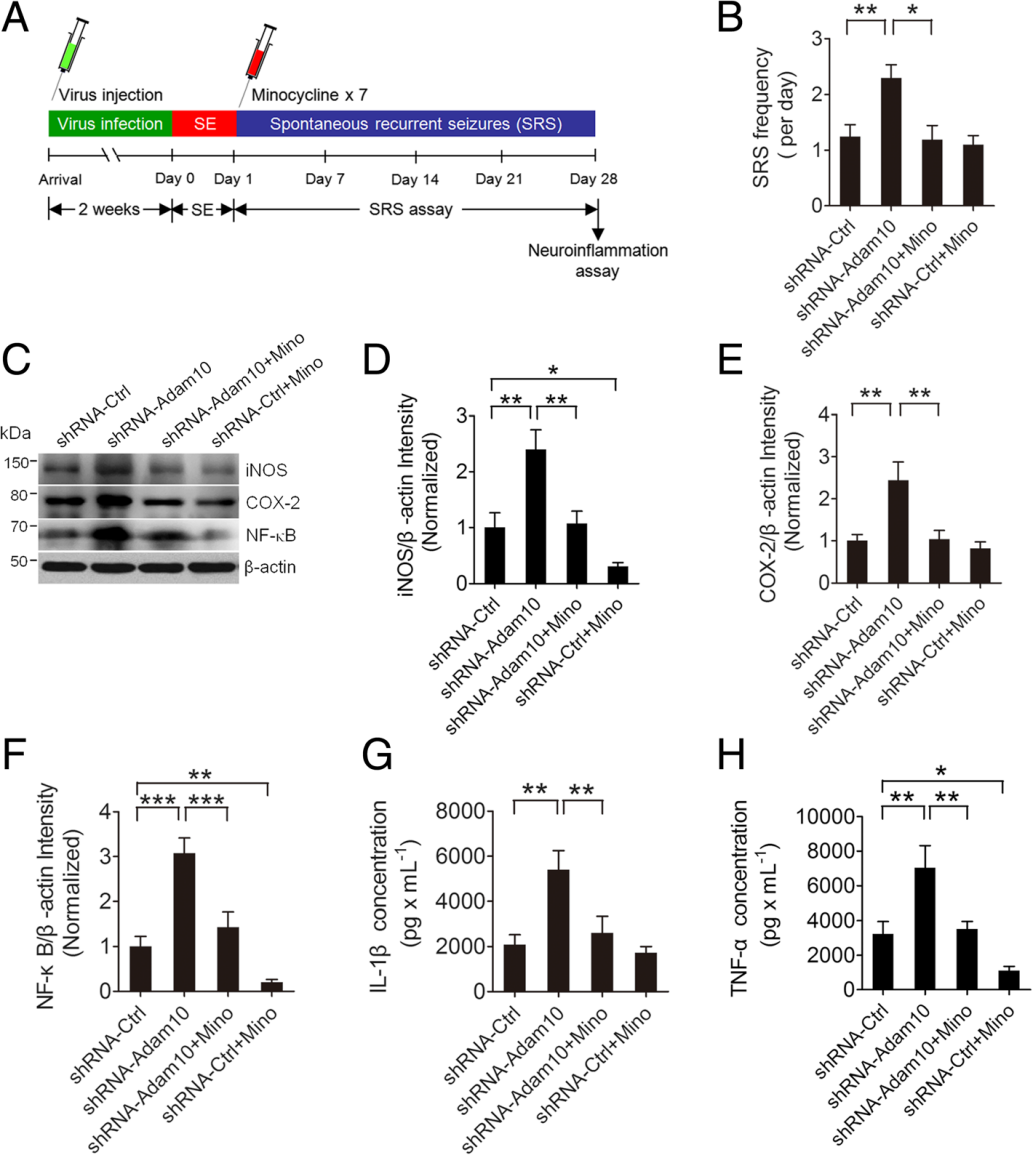
Increased seizure activity by Adam10 knockdown is dependent on hippocampal neuroinflammation. **a** Schematic diagram of the experimental design. Mice were bilaterally injected into the hippocampus with either Vehicle Ctrl or lentivirus carrying the shRNA-Ctrl or shRNA-Adam10. Following 2 weeks of recovery, the mice were induced to SE, and 24 hours after the SE induction, they were treated with minocycline (50 mg/kg, i.p.) seven times at 24-hour intervals. The mice were continuously video EEG monitored for 4 weeks for SRS analysis. The mice were then sacrificed after the EEG recording was completed at day 28 post-SE for analysis of hippocampal neuroinflammation. **b** Bar graph showing the SRS frequency in the shRNA-Ctrl, shRNA-Adam10, and shRNA-Adam10 + Minocycline- and shRNA-Ctrl + Minocycline-treated TLE mice. A two-way ANOVA revealed a significant main effect of Adam10 knockdown (*F*_1,20_ = 6.60, *p* = 0.02), minocycline treatment (*F*_1,20_ = 7.90, *p* = 0.011), and Adam10 knockdown × minocycline interaction (*F*_1,20_ = 4.69, *p* = 0.043) on SRS frequency. A Tukey post hoc test revealed that SRS frequency was significantly increased in shRNA-Adam10 mice compared to that in shRNA-Ctrl mice (*p* = 0.003). Minocycline treatment suppressed the shRNA-Adam10-induced increase of SRS frequency (*p* = 0.02), while the minocycline-treated shRNA-Ctrl mice did not show any significant difference of SRS frequency compared to the shRNA-Ctrl mice (*p* = 0.087) (*n* = 6). **c** Western blotting showing the protein levels of the inflammation-related proteins iNOS, COX-2, and NF-κB in the hippocampus of shRNA-Ctrl, shRNA-Adam10, and shRNA-Adam10 + Minocycline- and shRNA-Ctrl + Minocycline-treated TLE mice. **d**–**f** Bar graphs showing the quantification of iNOS, COX-2, and NF-κB as measured by the intensity ratios of these proteins to β-actin. For iNOS, a two-way ANOVA revealed a significant main effect of both Adam10 knockdown (*F*_1,16_ = 19.13, *p* < 0.001) and minocycline treatment (*F*_1,16_ = 16.67, *p* < 0.001) on iNOS protein level, but there was no significant interaction between Adam10 knockdown and minocycline treatment (*F*_1,16_ = 1.60, *p* = 0.224). A Tukey post hoc test revealed that the iNOS protein content was significantly increased in shRNA-Adam10 mice compared to that in shRNA-Ctrl mice (*p* = 0.001). Minocycline treatment suppressed the shRNA-Adam10-induced increase in iNOS protein level (*p* = 0.002), Moreover, the iNOS protein level in minocycline-treated shRNA-Ctrl mice was significantly decreased compared to that in shRNA-Ctrl mice (*p* = 0.044). For COX-2, a two-way ANOVA revealed a significant main effect of Adam10 knockdown (*F*_1,16_ = 9.98, *p* = 0.006), minocycline treatment (*F*_1,16_ = 9.05, *p* = 0.008), and Adam10 knockdown × minocycline interaction (*F*_1,16_ = 5.37, *p* = 0.034) on the COX-2 protein level. A Tukey post hoc test revealed that COX-2 protein content was significantly increased in shRNA-Adam10 mice compared to that in shRNA-Ctrl mice (*p* = 0.001). Minocycline treatment suppressed the shRNA-Adam10-induced increase in COX-2 protein level (*p* = 0.002), while the minocycline-treated shRNA-Ctrl mice did not show any significant difference in the COX-2 protein content compared to that in shRNA-Ctrl mice (*p* = 0.631). For NF-κB, a two-way ANOVA revealed a significant main effect of both Adam10 knockdown (*F*_1,16_ = 37.88, *p* < 0.001) and minocycline treatment (*F*_1,16_ = 20.67, *p* < 0.001) on the NF-κB protein level, but there was no significant interaction between Adam10 knockdown and minocycline treatment (*F*_1,16_ = 2.46, *p* = 0.136). A Tukey post hoc test revealed that the NF-κB protein content was significantly increased in shRNA-Adam10 mice compared to that in shRNA-Ctrl mice (*p* < 0.001). Minocycline treatment suppressed the shRNA-Adam10-induced increase in NF-κB protein level (*p* < 0.001). Moreover, the NF-κB protein level in minocycline-treated shRNA-Ctrl mice was significantly decreased compared to the levels in the shRNA-Ctrl mice (*p* = 0.008) (*n* = 5). **g**, **h** Bar graphs showing the concentration of IL-1β and TNF-α in the hippocampus of shRNA-Ctrl, shRNA-Adam10, and shRNA-Adam10 + Minocycline- and shRNA-Ctrl + Minocycline-treated TLE mice as detected by ELISA. For IL-1β, a two-way ANOVA revealed a significant main effect of both Adam10 knockdown (*F*_1,16_ = 11.14, *p* = 0.004) and minocycline treatment (*F*_1,16_ = 6.31, *p* = 0.023) on IL-1β concentration, but there was no significant interaction between Adam10 knockdown and minocycline treatment (*F*_1,16_ = 3.781, *p* = 0.070). A Tukey post hoc test revealed that the IL-1β concentration was significantly increased in shRNA-Adam10 mice compared to that in shRNA-Ctrl mice (*p* = 0.002). Minocycline treatment suppressed the shRNA-Adam10-induced increase in IL-1β concentration (*p* = 0.006), while the minocycline-treated shRNA-Ctrl mice did not show any significant difference in IL-1β concentration compared to that in shRNA-Ctrl mice (*p* = 0.693). For TNF-α, a two-way ANOVA revealed a significant main effect of both Adam10 knockdown (*F*_1,16_ = 16.09, *p* = 0.001) and minocycline treatment (*F*_1,16_ = 13.32, *p* = 0.002) on TNF-α concentration, but there was no significant interaction between Adam10 knockdown and minocycline treatment (*F*_1,16_ = 0.83, *p* = 0.375). A Tukey post hoc test revealed that the TNF-α concentration was significantly increased in shRNA-Adam10 mice compared to that in shRNA-Ctrl mice (*p* = 0.003). Minocycline treatment suppressed the shRNA-Adam10-induced increase in TNF-α concentration (*p* = 0.005). Moreover, the TNF-α concentration in minocycline-treated shRNA-Ctrl mice was significantly decreased compared to that in shRNA-Ctrl mice (*p* = 0.041) (*n* = 5). *p < 0.05, **p < 0.01, ***p < 0.001, and two-way ANOVA

Beyond its role as a pathological hallmark of epilepsy, we hypothesized that neuroinflammation could affect the process of Adam10-regulated epileptogenesis. To test this hypothesis, we treated Adam10 knockdown and control mice with pilocarpine to induce SE, followed by multiple doses of the anti-inflammatory agent minocycline to block neuroinflammation. All animals were subjected to continuous video EEG monitoring from the start of SE induction until 4 weeks post-SE (Fig. 9a). We then analyzed the SRS in these mice. Our results show that minocycline treatment significantly suppressed the Adam10 knockdown-induced increase of SRS (Fig. 9b).

To confirm the anti-inflammatory effect of minocycline, we used Western blotting to examine the hippocampal protein levels of the inflammatory mediators iNOS and COX-2 and the inflammatory transcription factor NF-κB after minocycline treatment. Our results reveal that minocycline treatment suppresses the Adam10 knockdown-induced increase in expression of the inflammatory mediators iNOS and COX-2 and the inflammatory transcription factor NF-κB (Fig. 9c–f). Furthermore, we have observed a remarkable reduction of iNOS (Fig. 9d) and NF-κB (Fig. 9f) expression after minocycline treatment in shRNA-Ctrl-treated mice. Consistent with the Western blotting results, ELISA reveals that minocycline suppressed the Adam10 knockdown-induced increase in the production of IL-1β and TNF-α (Fig. 9g, h). Moreover, minocycline treatment decreased TNF-α levels in ShRNA-Ctrl mice (Fig. 9h).

Taken together, these results suggest that increased seizure activity in the Adam10 knockdown TLE mice is dependent on hippocampal neuroinflammation.

## Discussion

Adam10 was initially identified as an alpha-secretase in the processing of the amyloid precursor protein, which is involved in Alzheimer’s disease. Recent studies shed light on the link between Adam10 and another neurological disease, such as epilepsy. Our findings that Adam10 is abundantly expressed in the hippocampal region highlight the importance of Adam10 for the regulation of neural activities in the hippocampus. The hippocampus is a region of the forebrain, which is highly vulnerable to excitotoxic injury and is largely involved in epileptic seizures. Therefore, it is plausible that the Adam10 gene regulates the development of epilepsy via modulation of hippocampal neural circuit activities. We have shown that Adam10 expression in the hippocampus progressively decreases from day 14 to day 28 post-SE. Consistent with our findings, a previous study reported that Adam10 mRNA levels were significantly downregulated in the CA1 and CA3 pyramidal cell layers of the hippocampus at 24 h after a kainic acid-induced generalized seizure [10].

Recent studies implicate neuroinflammation as playing a crucial role in the pathophysiological processes of both animal and human TLE [31–33]. It has been reported that neuroinflammation occurs following SE in rodent brains and is associated with the process of chronic recurrence of spontaneous seizures [34]. Here, we demonstrate that the inflammatory mediators iNOS and COX-2 and the transcription factor NF-κB in the hippocampus of pilocarpine-induced TLE mice are significantly increased, which is consistent with previous reports [35, 36]. Additionally, the proinflammatory cytokines IL-1β and TNF-α are increased as well.

Neuroinflammation in TLE mice is characterized by the production of inflammatory mediators and cytokines as well as glial activation [32, 37]. It has been reported that glia activation occurs following prolonged seizures and is considered to be involved in the subsequent proinflammatory cytokine production [34, 38]. Consistently, in this study, we found that both microglia and astrocytes are significantly activated in the hippocampus of TLE mice. It has been suggested that seizure activities lead to the production of proinflammatory mediators, such as IL-1β and TNF, which in turn affect seizure severity and recurrence [34]. Furthermore, systemic injection of lipopolysaccharide, an inducer of inflammation in the brain, increases the seizure susceptibility [39, 40]. In agreement with these studies, we find here that neuroinflammation in the hippocampus of TLE mice is accompanied by increased spontaneous seizure recurrence after SE. Combined with previous data, our findings imply that prolonged SE activates microglia and astrocytes and induces inflammatory mediators and cytokines, which may contribute to the increased spontaneous seizure recurrence in TLE mice.

Adam10 has been suggested to be involved in the neuroinflammation process under the conditions of epilepsy. Herein, we demonstrate that overexpression of Adam10 in the hippocampus suppresses neuroinflammation and reduces seizure activities, while inhibition of Adam10 exacerbates hippocampal neuroinflammation and increases seizure activity in TLE mice. Consistent with our findings, a previous study by Clement et al. reported that overexpression of Adam10 decreased seizure activity and suppressed neuroinflammation by reducing glia activation in a kainate-induced seizure model [41]. Interestingly, Clement et al. also demonstrated that when there is a lack of APP expression, overexpression of Adam10 leads to increased neuroinflammation and seizure activity [41]. These findings suggest that the action of Adam10 may be dependent on its substrates. To further investigate whether the effect of Adam10 on seizure activity is dependent on hippocampal neuroinflammation in TLE mice, we induced SE in Adam10 knockdown mice, followed by the treatment with the anti-inflammatory agent minocycline. We demonstrated that minocycline treatment suppressed the Adam10 knockdown-induced increase of spontaneous recurrent seizures. Minocycline is known as an inhibitor of microglial activation which selectively inhibits microglia-related gene expression [42]. Therefore, it is possible that minocycline suppresses seizure activity in Adam10 knockdown mice through repression of microglia-mediated neuroinflammation.

## Conclusions

Our data identify Adam10 as a key regulator of hippocampal neuroinflammation-dependent seizure activity in pilocarpine-induced TLE mice. Our results suggest that the modulation of hippocampal neuroinflammation via Adam10 could play a pivotal role in the development of epilepsy.

## Abbreviations

AAV: Adeno-associated virus
AD: Alzheimer’s disease
Adam: A disintegrin and metalloproteinase domain-containing protein
Aβ: Amyloid β
ELISA: Enzyme-linked immunosorbent assay
sAPP: Soluble N-terminal APP fragment
SE: Status epilepticus
TLE: Temporal lobe epilepsy
